# "We need time, a great know-how and security for patients to always be there in time”: a qualitative study on factors distinguishing General from Specialized Palliative Home Care

**DOI:** 10.1186/s12913-025-12258-y

**Published:** 2025-02-13

**Authors:** Melanie Joshi, Kim Dillen, Norbert Krumm, Michaela Hesse, Holger Brunsch, Julia Strupp, Lukas Radbruch, Roman Rolke, Raymond Voltz, on behalf of the APVEL Consortium, Lukas Radbruch, Roman Rolke, Raymond Voltz, Holger Pfaff, Nadine Scholten, Martin Hellmich, Christian Rietz, Ingo Meyer, Robin Fink

**Affiliations:** 1 Faculty of Medicine and University Hospital, Department of Palliative Medicine, University of Cologne, Cologne, Germany; 2 Department of Palliative Medicine, Medical Faculty RWTH Aachen University, Aachen, Germany; 3 Institute for Digitalization and General Medicine, RWTH Aachen University, Aachen, Germany; 4 Department of Palliative Medicine, University Hospital Bonn, Bonn, Germany; 5 Centre for Palliative Medicine, Helios Hospital Bonn/Rhein-Sieg, Bonn, Germany; 6 Faculty of Medicine and University Hospital, Center for Integrated Oncology, Aachen Bonn Cologne Duesseldorf (CIO ABCD), Aachen, Germany; 7 Faculty of Medicine and University Hospital, Center for Health Services Research, University of Cologne, Cologne, Germany; 8 Faculty of Medicine and University Hospital, Center for Integrated Oncology, Aachen Bonn Cologne Duesseldorf (CIO ABCD), Cologne, Germany

**Keywords:** Palliative home care, Qualitative interviews, Healthcare professionals, General palliative home care, Specialized palliative home care

## Abstract

**Background:**

An increasing number of patients in the palliative phase of their disease are cared for at home by palliative home care services. A sense of security, normality of everyday life and symptom control are found to be active factors of quality of care in Specialized Palliative Home Care. Whether this also applies to General Palliative Home Care has not yet been systematically investigated. The aim of this study was to identify distinctions between General and Specialized Palliative Home Care from a healthcare professional’s perspective concerning those factors.

**Methods:**

With a qualitative approach, we conducted 11 semi-structured interviews with healthcare professionals from different professional backgrounds in General and/or Specialized Palliative Home Care.

**Results:**

In both General and Specialized Palliative Home Care, healthcare-professionals (HCP) found a *sense of security* (through *availability*) to be most relevant for the patients. The majority saw aspects of *normality of everyday life* as a key component for high-quality palliative home care, especially *having time* for the patient and the family caregiver(s). However, statements about *symptom control* are mainly related to Specialized Palliative Home Care. The subcodes *availability*, *having time* and *competence, symptom burden* and *financial resources* were the main distinguishing factors between General and Specialized Palliative Home Care in *sense of security*, *normality of everyday life* and *symptom control,* respectively.

**Conclusions:**

Our results provide the basis for a clearer definition of GPHC and SPHC and contribute to identifying factors for a transferal between the two services to provide best care for the patient. Distinguishing (sub)factors revealed challenges and short-term solutions. Providing (financial) incentives to guarantee time and availability in General Palliative Home Care would lead to more effective care.

**Supplementary Information:**

The online version contains supplementary material available at 10.1186/s12913-025-12258-y.

## Background

Internationally, the preference to die at home remains high and is even increasing [1–9], highlighting the importance of palliative home care which enables patients to stay at home and be cared for in their preferred place until they die. In Germany, this preference is answered—alongside standard homecare—by palliative home care through a system with two types of care-services: General Palliative Home Care (GPHC) and Specialized Palliative Home Care (SPHC).

GPHC can be performed either by a registered physician with own practice, usually a general practitioner (GP) with basic palliative qualifications, or by palliative homecare-teams consisting partly of nurses with basic palliative training who collaborate with registered physicians with their own practices and basic palliative qualifications or a registered palliative specialist [10]. In distinction, a SPHC-team is defined through its multi-professionalism and physicians and nurses working in SPHC require a specialization in palliative medicine or care [11–13].

Internationally, the view on palliative home care has mainly been described through the eyes of patients, family caregivers, GPs or district nurses, taking place in settings other than homecare, in just one group of professionals or in General and Specialized Palliative Home care, separately [3, 14–19]. Since there are various professions working in palliative home care services, it is of utmost importance to include professions other than nurses and physicians to reflect their (different) perspectives on their daily work. Nevertheless, qualitative findings from the perspective of healthcare professionals, especially on interviewing multiple professions in one study in the field of palliative home care are scarce.

Distinctions have been described between primary care and specialized (outpatient) care only [20] and we found only one study discussing distinctions between GPHC and SPHC within a homogeneous group of physicians [21]. While the performance of SPHC has been evaluated in a previous study in Bavaria, Germany, finding three active factors—*sense of security, normality of everyday life* and *symptom control* [22], whether these factors apply to GPHC and what (sub)factors distinguish GPHC and SPHC has not been systematically investigated.

The aim of our study was to identify (sub)factors of those active factors that distinguish GPHC and SPHC from the perspective of multi-professional healthcare professionals. To the best of our knowledge, this is the first study on distinguishing factors between the two types of palliative care services in palliative home care focusing on those active factors. Knowledge of distinguishing factors would provide additional insight into appropriateness of the level of care for patients. This may contribute to a clearer definition of GPHC and SPHC and help to identify factors for transfer between the two services in future research.

## Methods

### Aim

The study was part of the project “**A**mbulante **P**alliativ**V**ersorgung **E**va**L**uieren” (APVEL) in North Rhine, Germany with the aim to evaluate SPHC in contrast to GPHC in this region analyzing the perspectives of patients, family members and healthcare professionals. The study was conducted in collaboration with the Departments of Palliative Medicine in Aachen, Bonn and Cologne [23]. Data on patients and family members are published elsewhere [24].

### Setting and sample

Healthcare professionals were recruited in two different types of care-services (SPHC, GPHC, or both). In order to gain a differentiated picture, different professional perspectives on the field of palliative home care were needed to reflect the multi-professional reality in this field (see Table 1).

**Table 1:** Profession, type of care service and gender of healthcare professionals (*n* = 11)

Profession	Type of care	Gender	N
Nurse	SPHC	m	1
		f	1
Nurse	GPHC	m	1
		f	1
Manager of a care service	GPHC	f	1
Social worker	GPHC	f	1
Ecotrophologist	SPHC/GPHC	f	1
Physician	SPHC/GPHC	m	2
		f	2

### Characteristics of participants

Physicians and nurses usually treat symptoms, e.g. pain and nausea, inform patients (and caregivers) on medical treatment and processes of treatment or body functions. Ecotrophologists consult on nutrition tailored to the patient’s condition and/or tube feed. Managers of care services organize team structures, hold an overview over (potential) cooperating partners and treated patients, whereas social workers usually help patients (and caregivers) with burocratical, financial and organizational tasks, e.g. change of care setting and care aids. Within this particularly narrow field of research and the places were the study was conducted, were only very few Palliative Home Care Services consisting of very few persons. So, we decided to not collect age or work experience, since this may lead to de-anomyzation of participants.

### Data collection

The semi-structured interviews of healthcare professionals in both settings (*n* = 11) took place during March 2018 until November 2018. A purposive sampling design was used to meet the goal to interview different professions in GPHC and SPHC. All participants taking part in the study were approached via telephone and informed about the study, gave written informed consent and accepted the audio-recording of the interview, which took place on the premises of the interviewing clinic or the participant’s own office or practice without the presence of non-participants. NK was known due to his former clinical work in Aachen, but participants in Bonn and Cologne had no prior relationship to or knowledge of the interviewer. There was also no refusal to take part in the study, drop-out during the study or repeat interviews.

Before the interview-phase started, we held a workshop with all interviewers to align our interview technique and had constant contact whenever questions concerning data collection arose throughout the study. KD (f), MJ (f), MH (f), HB (m) and NK (m) performed the interviews by using an interview guide which had been created following three active factors—*sense of security*, *normality of everyday life* and *symptom control*—identified in a previous qualitative study with SPHC-teams, patients and their family caregivers [25], additionally asking for experienced differences between General and Specialized Palliative Home Care. Field notes were taken directly after each interview took place.

The different professional backgrounds of all researchers enriched interviews and analysis and increased reflexivity out of a multi-professional perspective; KD has a background in psychology (PhD), MJ holds a degree in humanities and social sciences (M.A.), NK in Research (M.Sc.) and MH in Palliative Care (PhD), HB is a historian (PhD) and MJ, NK and MH are registered nurses. KD, MJ, HB and NK were full-time researchers, while MH also worked as a case manager at a palliative care unit. All researchers had an interest in the topic because of their professional background in patient care (KD, MJ, NK, MH) or methodology (HB), or both. Two researchers had a migration background. Outcomes in this article are reported in line with the consolidated criteria for reporting qualitative research (COREQ) guidelines.

### Data analysis

The audio-recorded interviews were transcribed verbatim using Lamnek’s principles of transcription [26]. Transcripts were not returned to participants for correction nor for feedback to maintain the originality of the interview. Qualitative content analysis was used to structure the text [27, 28]. The overall codebook including all relevant codes was developed inductively by MJ, who, at the time, was not familiar with the topic, based on five interviews finding main codes (*n* = 7) and subcodes (*n* = 20). The independent verification of the codes by a second researcher (KD) confirmed the relevance and accuracy of the codes. All interviews were analyzed by KD, MJ, NK, HB and MH utilizing the qualitative data analysis software MAXQDA 18 [28].

In a further step, all interviews were re-coded by a second person in the local research team, leading to discussions on the codebook first in the local team, then in regular meetings during the coding and re-coding process of the research group. Any discrepancies led to a more precise differentiation of the definitions and required minor extensions of the codebook due to the addition of further inductive subcodes due to different perspectives of the professional groups on palliative home care. We also created a code *distinguishing factors* and added it to any relevant coding to be able to identify the most important distinguishing factors within the overall codes.

The Code Relations Browser was used for clarification of overlapping *distinguishing factors* and the three active factors *sense of security*, *normality of everyday life* and *symptom control* and their subcodes*,* respectively*.*

## Results

**Fig. 1:**
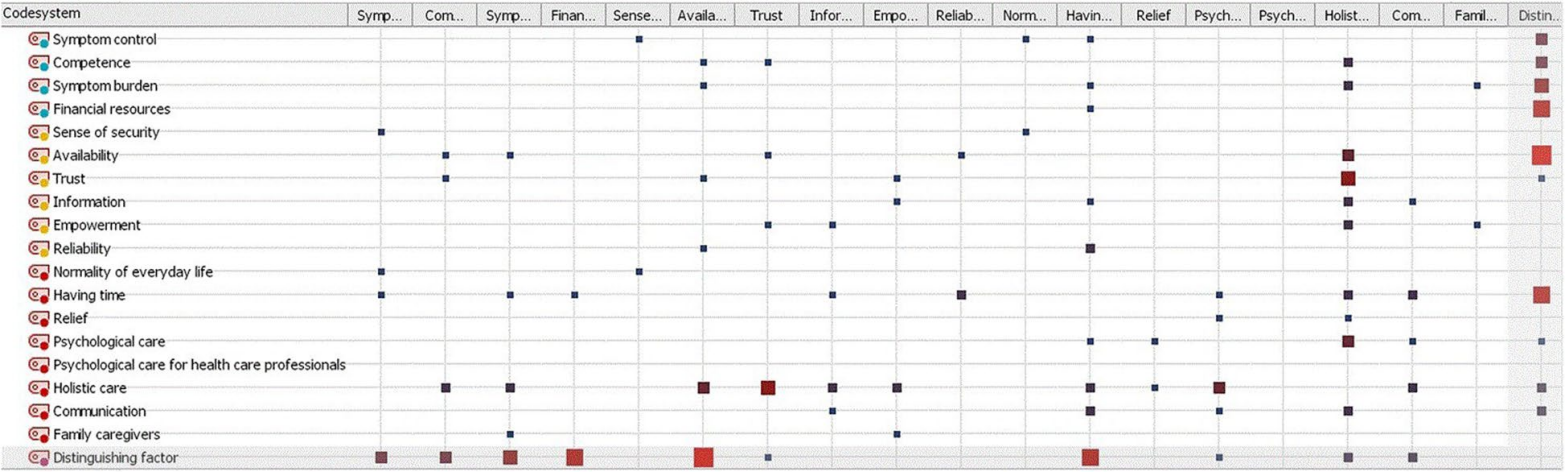
Code Relations Browser showing the frequency of overlap between the code *distinguishing factor* and the (sub)codes of the three active factors the interview guide was based on. First line presents the same codes as first row. Larger squares symbolize a higher relation between codes. MAXQDA presents the codes and subcodes on an equal level, but the codes *sense of security*, *normality of everyday life* and *symptom control* are higher-level codes

The average interview length was 25 min, differing in range between 15 and 62 min. All three active factors showed aspects of distinction between GPHC and SPHC explained by at least one subcode of our findings. The following subcodes indicate distinguishing factors in palliative home care (Fig. 1).

### Sense of security

If healthcare professionals are able to assure a comprehensive security at home in a credible and dependable way, patients (and their family caregivers) gain a *sense of security* [29]. Our results show a consensus from all respondents that a *sense of security* reached through *availability* being most important for the effectiveness of palliative home care.

“The patient must have the feeling - I have heard it again and again - that we [patient and relative] are at home, not in the hospital, but we can always call someone and we know who is coming. I think that is a very important track.”

(Social worker, GPHC, HCP11)^1^

### Availability as distinguishing factor

*Availability* was guaranteed by giving out only one telephone number that made it easy for the patient to reach a healthcare professional while team members passed on the phones depending on office hours and on-call duty. Different systems that have been established to guarantee *availability* were described, e.g., on-call duty within a team or the connection of GPHC services to SPHC on-call duty, which then also covers the GPHC patients. However, the physicians particularly emphasized the fast and immediate *availability* of the SPHC teams in distinction to GPHC and being a substitute to emergency services:

“So, when they call, you are at the home visit within ten minutes and this TOTAL security, which we give them, within SPHC more timely, but to also be always available in GPHC, that is a good palliative care in my eyes […] and gives the family members, the patients the security, they can ALWAYS not call [the emergency number] 112, but we are there.”

(Physician, GPHC/SPHC, HCP05)

Participants distinguished between *availability* of the palliative home care-team itself for patients/family caregiver and the *availability* of physicians for nurses in GPHC.

Nurses emphasized the importance of reliable cooperation, when describing situations where availability was not guaranteed by collaborating medical staff in GPHC. Reasons were either a time where GPHC-nurses were not able to reach the registered physician beyond office hours or if they were not dependable (in Germany, only physicians are allowed to prescribe medication):

"They always say: ‘Mrs. <NAME OF THE MANAGER> we need painkillers. We are not physicians; the physician is not available. We are standing here. What shall we do? We are not allowed to do this and that. What shall we do?’"

(Manager of a care service, GPHC, HCP06)

However, solution strategies were also highlighted, e.g., resident physicians prescribing on-demand medications in advance, which could then be used by the GPHC-nurse in an emergency or in the terminal phase and avoids having to call the emergency services.

Participants, especially physicians, who decide on the type of care in Germany, see the frequency of providers having to be available, as indicator of a change in care and *distinguishing factor* of SPHC and GPHC:

“So, I think if a patient needs to be seen at home MORE than once a week, then you have to think about whether SPHC co-care is needed. And a patient is SURELY SPHC if they need to be seen by a PHYSICIAN once a week, plus care and a patient is SURELY SPHC if a specialized nurse or a physician needs to come three times a week […] It does have something to do with the frequency or the frequency of calling for nursing or medical services.”

(Physician, GPHC/SPHC, HCP07)

### Normality of everyday life

The act of awareness of the palliative home care-teams to adapt to the framework of everyday life in the patient’s home and bring normality into the exceptional situation of dying is described *as normality of everyday life* [22]. *Holistic care* is mentioned by the majority (81%) of the participants by speaking about the patient’s will and personal wishes, e.g. enable social events, engage volunteer-services and deal with the patient’s social surroundings:

“And he [healthcare professional] goes there promptly and tries to find out the situation: Does he [the patient] need a pump? Can we still give the medication on the spot, is it still enough? Does he need other care? Is it more the wife or husband or spouse who can't take the situation well?”

(Physician, GPHC/SPHC, HCP05)

In addition*, having time* is described as precious by the majority (73%) of the participants in the sense of being able to have longer conversations with patients and family caregivers and being there for the patient without time pressures.

“TIME is one thing, because it takes time to get a family back on board and to have these conversations and also to repeat the seventh time, or the tenth time, why water at the end of life is absolutely not important, why nutrition is no longer so important at some point […]”

(Physician, GPHC/SPHC, HCP05)

### Having time as distinguishing factor

Not only does *having time* play a major role in the vision of good care in the health sector, where usually time is scarce, but at the same time serves to highlight differences between GPHC and SPHC:

“The problem […] is that we simply don't have time for the patients. I make ten house calls in two hours lunch break and in SPHC I make eight house calls in eight hours. That's a huge difference. “

(Physician, GPHC/SPHC, HCP08)

In addition, the flexibility in palliative home care is described by *having time*:

“Part of your attitude is that you are available for the patients and try to take the time despite a busy practice. And where the time is too much, too time-consuming, I hand over to SPHC.”

(Physician, GPHC/SPHC, HCP05)

Although time is seen as lacking in GPHC in comparison to SPHC, the participants describe trying to give the patients/family caregiver the time they need:

“So my nurse doesn't leave when the relative cries or she sees, I have to stay there. I only get sum X and closing time, no matter how long I'm there, but I don't demand ‘thirty minutes, you have to go back’ from my co-worker. We don't do that.”

(Manager of a care service, GPHC, HCP06)

Nevertheless, some interviews shed light on how participants defined the palliative home care-type in comparison to (non-palliative) standard homecare, showing their satisfaction about *having time* in their field of work.

“So, time, that is really the key word for us […] And I also feel that this is a luxury good compared to the girls who work in the standard nursing service. They work so hard, they do a great job and I think they're also super emphatic. But they always have to work with such time pressure.”

(Nurse, GPHC, HCP03)

“And a normal nursing service simply can't do that. And that is why these SPHC services, which can then also take time, can sit down with the patient. They can also talk to the patient about the situation or perhaps also find solutions for the relatives, not only for the patient in the context of pain therapy, but also care for the relatives.”

(Ecotrophologist, GPHC/SPHC, HCP01)

### Symptom control

While there are many definitions of the term symptom and as many approaches to the term symptom control [25], the layperson uses the term for the control of physical symptoms such as pain or breathing difficulties. For our analysis, we have used a definition that also includes psychological aspects. The frequency of participants describing *symptom control* (46) was not very high compared to *sense of security* (84) and *normality of everyday life (134)*. However, *competence, symptom burden* and *financial resources* were subcodes of *symptom control* holding distinguishing characteristics between GPHC and SPHC.

### Competence, symptom burden and financial resources as distinguishing factors

Participants working in both types of care-services describe *competence* and *symptom burden* as *distinguishing factor*s. In their opinion, SPHC provides and has to provide more intense care, because of the complex *symptom burden* their patient population experiences.

Therefore, SPHC-teams have and need to have high *competence* controlling symptoms. *Competence* was seen as theoretical knowledge and/or practical know-how used directly on the patient, being seen as more intensively required in SPHC, where *competence* also meant different competences in different fields within a team, highlighting multi-professionalism:

“SPHC is care by specialists, GPHC is intensified care by GPs. And in the [specialized] palliative area it is simply one step further, because you can carry out other symptom controls, other wound care and also other psycho-oncological care.”

(Physician, GPHC/SPHC, HCP10)

In addition, patients in SPHC are distinguished from GPHC patients regarding their *symptom burden*:

“A typical SPHC patient is a patient who has a high level of suffering, i.e., a severe symptom burden, which is also one of the criteria that is important for admission.”

(Physician, GPHC/SPHC, HCP08)

Participants of both types of care-service mentioned the experience of inequality in the distribution of *financial resources* in palliative home care. SPHC-patients benefitted from a system, where a certain financial leeway is given, e.g., being able to prescribe individualized medication, which is highly effective, but comes with higher costs or being able to contact a patient with *symptom burden* with higher frequency than in GPHC. HCP felt that the financial conditions led to unjust care circumstances in terms of time with impact on the patient as well as the physician.

“[…] one can't afford to talk to five different relatives in the practice [as registered physician in GPHC], or I can't afford to do conferences for a GP, to be honest, it's illusory if you want to run a business economically.”

(Physician, GPHC/SPHC, HCP08)

Lacking *financial resources* in regard to *symptom control* in GPHC is seen as a reason to admit the patient to SPHC:

“We can't get that financed, so it has to go to the SPHC. And actually, I think that's a good thing. Sometimes I think a patient could have been transferred to SPHC earlier.”

(Social worker, GPHC, HCP11)

Overall, participants viewed both systems as independent, while negative parts of the system were emphasized more often in GPHC, revealing challenges and *distinguishing factor*s. However, the flexibility of the system was emphasized, as were the possibilities to refer patients whose condition improved or deteriorated into the other system, respectively.

## Discussion

We found that healthcare professionals rate a *sense of security* and *normality of everyday life* as most important in both types of care services, with the subfactors *availability* and *having time* and *holistic care*, respectively. The frequency of participants describing *symptom control* was not very high compared to *sense of security* and *normality of everyday life,* which can be explained by the situation where some professions had a different focus than noticing or treating symptoms. Although *symptom control* was mainly referred to by healthcare professionals working in SPHC, *competence, symptom burden* and *financial resources* were *distinguishing factor*s between GPHC and SPHC.

Our results show that *availability* gives a *sense of security,* which was critical for all healthcare professionals taking part in the study. This is in line with the patient’s and family caregiver’s view in palliative home care [18, 22, 24, 25, 29, 30], evaluation of the effectiveness of healthcare professionals in SPHC [31] and GPs [21]. Although most telephone contacts take place mainly to find solutions to organizational problems rather than emergencies in palliative home care [32], the mere ability to contact a professional in the case of an emergency should not be underestimated for patients and family caregivers to feel secure.

Our interviews highlight a distinction between the way and means of *availability*, which arises between GPHC and SPHC. Whereas SPHC is described as fast in the sense of timeliness, as mentioned in a study with several specialized care teams [31], steady availability (beyond office hours) is seen as crucial for GPHC. In GPHC, not being able to contact the registered physician with their own practice is described as a burden to nurses and was also reported in a Norwegian study interviewing GPs and home care nurses [3], leading to a less effective care.

The importance of *having time* to establish a *normality of everyday life* for the patient and to identify palliative care needs is consistent with previous studies in palliative settings [3, 18, 33]. *Having time* was compared to different care systems: SPHC physicians typically described the leeway of time they have in comparison to GPHC, whereas other professions referred to having more time than in standard homecare. As highlighted in a previous study, SPHC is particularly indicated when care requires such a large amount of time that it cannot be provided through a GP [29], which can be confirmed by our interviews emphasizing the flexibility of the two-path care system in those situations.

*Holistic care,* aimed at the patient’s psychosocial, spiritual needs as well as the physical ones, is a main characteristic in palliative care [34, 35], so it was not surprising that the majority find holistic care to be important for effective care and it does not serve as relevant *distinguishing factor*.

*Competence*, verified through certified specialized palliative (care) training is a legal directive required for SPHC [13]. Nevertheless, *competence* was also defined in SPHC as multi-competence while working in a multi-professional team.

Since most patients in the palliative phase of their disease experience symptoms like pain, dyspnea or nausea, *symptom control* (or symptom management) is an often mentioned criteria for quality in palliative care based on the high *symptom burden* of patients in palliative care [5, 18, 34]. However, its need depends on the severity and complexity of the patient’s *symptom burden*. In Germany, a complex *symptom burden* serves as a criterion which patients in SPHC have to fulfil, thus explaining why *symptom burden* serves as a *distinguishing factor* [13, 22].

A lack of *financial resources* is found to be a barrier for symptom management related to psychosocial and spiritual care [36, 37]. Our results show that it is also linked with physical treatment, e.g., a leeway to prescribe individualized medication.

### Limitations

By using qualitative methods with a deductive-inductive approach, we were able to build on existing knowledge and be very specific in a narrow field of research deductively based on a previous study, but also to find distinguishing factors inductively. The main findings are consistent throughout both types of care-services, achieving saturation. The strength of the study is that the sample consists of participants working in both palliative home care types existing in Germany and in a range of professions that reflect the reality in palliative home care.

A limitation of this sampling is that not all participants worked in a profession related to active symptom management, e.g. the perspective of a social worker on patients’ symptom burden may differ compared to a nurse or physician, which may explain the low reporting on symptom burden in this sample. With another sampling approach including only physicians and nurses, this category may have higher consensus and frequency.

Due to a rather small sample with a regional approach, the results are not representative of other palliative care settings or healthcare systems. However, a two-path system for palliative home care, including a general and a specialized structure, has also been established in other European countries, e.g., Switzerland [38] and Norway [30, 39], so comparability is given within comparable healthcare systems internationally.

## Conclusions

From a healthcare professional’s perspective, to ensure effectiveness in both settings of palliative home care, the guaranteed availability of physicians and having time for the care of patients and relatives is crucial. Individual solutions for challenges, e.g., giving time without being reimbursed for it or finding committed physicians who are available for GPHC nurses beyond office hours, tend to work as a short-term solution. Nevertheless, as a long-term solution, increasing financial resources and providing incentives to guarantee more time and availability in GPHC would lead to more effective care and patient satisfaction.

## Supplementary Information

Supplementary Material 1.

## Data Availability

The dataset generated and analyzed during the current study is not publicly available due to the limitations of ethical approval involving the data and anonymity but are available from the corresponding author upon reasonable request.

## Abbreviations

APVEL: Ambulante PalliativVersorgung EvaLuieren
COREQ: Consolidated Criteria for Reporting Qualitative Research
GP: General Practitioner
GPHC: General Palliative Home Care
HCP: Health-care professional
SPHC: Specialized Palliative Home Care

## Footnotes

1 To give information on the participant, we added profession (here: social worker), type of care service (here: General Palliative Home Care) and interview pseudonyme (here: HCP11).
