# Supplementary material for: "We need time, a great know-how and security for patients to always be there in time”: a qualitative study on factors distinguishing General from Specialized Palliative Home Care
Source: BMC Health Serv Res. 2025 Feb 13;25:245. doi: 10.1186/s12913-025-12258-y (PMC11823236; doi:10.1186/s12913-025-12258-y)
Supplement: Supplementary file 1 — Supplementary Material 1. [file 12913_2025_12258_MOESM1_ESM.pdf]

Annex I

Interview Guide

Starter question (HCP in SPHC)

- What constitutes SPHC work for you? What features characterize an SPHC case compared to a GPHC case?
- How is the GPHC/SPHC collaboration structured? What is the typical transition to SPHC like? How do you handle stabilizations? How would you describe the distinction from GPHC?

Starter question (HCP in GPHC)

- How has palliative home care evolved since the implementation of SPHC? From your point of view, what has changed for you as well as for the patients?
- How is the collaboration between GPHC and SPHC? What is the typical transition to SPHC like? How would you describe the distinction to SPHC?

(All HCP)

- What do you think is important to patients about home care? How do you notice this?

Symptom relief (physical symptom control)

- What is medical/nursing care like in a home setting?
- Who or what do you think is most relevant to successful relief of physical symptoms?
- What do you think are inhibiting factors for successful symptom relief?

Sense of security

- What are the overall services/people taking care of a SPHC/GPHC patient? What distinguishes them in particular?
  -Which of the existing care options do you think are most targeted?
- How does home-based palliative care create a sense of security for patients and why?

Normality of everyday life

- Is it easy for you to maintain the patient's home habits? Under which conditions is this particularly difficult/easy for you?
  -What, in your opinion, is particularly important to relieve the patients and, if applicable, their relatives in their everyday life?
- Are specific wishes/requirements of the patients taken into account? Can you briefly outline this with an example?
-Are you given enough time to take care of all the patients' needs in peace and quiet?
-How does the SPHC/GPHC team handle acute tensions or problem situations?
-Are there situations in which you are unable to communicate openly with patients?

Conclusion

- Now we have discussed a few things. Is there anything else you would like to share that is important to you but has not come up here in this interview?
- Do you have any questions? How was the interview for you?
